# Cancer of the Uterus*Read before the Clinton County Medical Society, held in Plattsburg, Mo., August 1, 1911.

**Published:** 1914-02

**Authors:** H. C. Crowell

**Affiliations:** Kansas City, Mo.


### Cancer of the Uterus.*

BY H. C. CROWELL, M. D., KANSAS CITY, MO.

When I was asked to present a paper before an open meeting of this society, I felt complimented and began to reflect upon what might be the most profitable and necessary subject for those not engaged in the practice of medicine. In going over my experience of thirty-six years of practice, I can think of nothing so important to the laity as the subject of cancer, particularly as it pertains to women, as I shall hope to show. So frequent and so on the increase is cancer that the profession as a whole are agreed that the greatest degree of light should be shed upon the subject, not only within our ranks, but that it should be brought home to the laity so often and so forcibly that they may not neglect, as now, those early manifestations, but seek assistance, while yet there may be a chance for its complete eradication.

We regret that we are obliged to admit that there are those, in the profession, who are not alert in recognizing the early manifestations of cancer, even in those who do present themselves for advice or treatment. Such men resort in a careless sort of way to the use of douches early, and later, when pain occurs, to the use of opiates until too late for anything except palliative treatment.

Some look upon cancer as incurable at any stage, and do little or nothing, though they may have recognized it. We would like to start out with the assertion that cancer is curable, if recognized in its inception, and treated, radically, by extirpation. We recognize the natural inclination to shrink from an operation

*Read before the Clinton County Medical Society, held in Plattsburg, Mo., August 1, 1911.

until it becomes a last resort procedure. When we contemplate, according to Crile, that, in this country alone, 80,000 people, now believing themselves well, will, in six months, have cancer, we can form some idea as to its frequency. It is not too much to say that there are those here tonight who have cancer and are either ignoring its symptoms or are ignorant in regard to them.

In view of such a conception of the subject, it is well for us to consider together those manifestations which should at least suggest attention, to the end of ascertaining the true character of such disturbances. Education of the people in regard to this subject has been too long neglected from various reasons, principally, perhaps, because of false modesty. Certainly not because of any fear on the part of the physician of losing business by such information, for no physician wishes to cope with cancer. On the contrary, information would add to one's clientele if the informed did justice to themselves, which is not always the case.

In speaking, as we shall at this time, of cancer in women, we would advert, in yet plainer terms, that statistics go to show that one in fourteen die of cancer, and that after the age of 39, one in nine. With this sordid picture presented, I am aware of the thoughts in your mind, naturally. What is the cause? Of this we are obliged to admit we can say, little. Research, experiments and observations have yielded nothing which has stood unvarying tests. More has been thought to be established in the line of causative factors, and yet on these lines we may be in error. However, it is accepted by most authorities that some things should be done along preventive lines. As, for example, if the cervix be torn in childbirth, it is thought that the neglected scar tissue is more likely to become the site of cancer, and, therefore, should be repaired. This fact and necessity is not as fully appreciated by the profession as it should be, and, if not by the profession, certainly the people will not give it the attention which they should. It is agreed, then, that all lacerations of the cervix are dangerous, and should be repaired. If neglected, and cancer does occur, to offer any chance or hope of success, it must be recognized early.

The early symptoms consist of a purulent vaginal discharge, bloody discharge it may be, or, more noticeable, should be any deviation from the normal of menstruation as to time or quantity. Pain may be said to be a later symptom of cancer, and its absence in no way proves that cancer is not developing.

We must urge it upon women to appreciate the importance of an early diagnosis, for upon it their life depends. We operate on late cancers, it is true, but with little hope of a permanent cure, but as a palliative means to lessen late suffering, and the removal of an offensive organ, often prolonging life a few months or years.

It is true that it is not always easy or possible for the average physician to tell whether a given case is in the beginning of cancer. In such case, when in doubt, the matter should be referred to one more skilled or better prepared to make such examinations. Usually, only the microscope will tell the story after a section of the affected cervix has been snipped out, or a scraping obtained from the inside of the uterus. Though such examination should prove negative, it is justifiable. We recognize that the word cancer pronounced to a patient, to them at least, spells death. As most often met, as we have said, in the neglected late stages, it is truly bad. But if we can secure an early diagnosis, such as we are praying for, it by no means needs be fatal. The whole medical fraternity the world over are agreed that cancer can only be lessened and lives saved by the people becoming more awake to its frequency and the need of their knowing more of its early symptoms in order that they may the earlier seek proper attention while yet there is a chance. See result of Winter's work in Germany.

We speak at this time, particularly, of cancer of women, since it is more frequent in the female, it being as 67 in 100, while in the male it is 38 to the 100, or nearly twice as common in the female as in the male. The most alarming feature of it all is the constant increase. The highest mortality of cancer in the female is between 45 and 60 years of age—in the male from 60 to 65. It is hardly necessary to say that the larger number of cancers occurring in women are of the uterus, but statistics are made for cancer including all sections where it may occur, as it does in the stomach (rather frequently), intestines, mouth, skin, breast, etc. In the male, cancer of the mouth and skin is more frequent than in the female. Cancer of the intestines is about equal in both male and female.

We have indicated that the most common age at which cancer occurs in women is 45 to 60. It is well known by women in general that the change of life, or menopause, occurs at from 38 to 50 years of age. This period is a critical one or may be, and should be viewed with observing care; any deviation from the usual, such as excessive or too frequent flowing, demands attention, although only a functional disturbance be found. Such disturbances may be the very early manifestation of malignancy.

Irritating, foul discharges should demand an examination and treatment. The most common error into which women fall is to think, with the recurrence of a bloody discharge after menstruation has ceased for several months or a year, that a recurrence of the menstruation is an evidence often to them of renewed vigor. No greater error is possible, for it almost universally means a beginning, or even well advanced malignancy. Too much emphasis cannot be placed upon this subject, for upon it hang the lives of thousands.

In this connection, we must decry the too frequent prudery, or false modesty, which keeps women from consulting a physician. It is proper, it is necessary, it is the only way to ascertain what these symptoms mean, and may be the means of saving a life. If such examination results in a negative, or uncertain conclusion, means should be taken to remove any doubt. It is no unjust criticism to say that the average general practitioner is hardly in a position to pass definitely upon such a case. It requires the trained eye of the pathologist to determine upon anything but the very advanced, or gross lesions, then too late to extend much, if any hope of permanent relief.

Turning from cancer of the uterus, we must remind you of the great frequency of cancer of the breast. Any lump in the breast should be considered worthy of attention, as it may be malignant, or soon become so. Operations properly performed for malignant disease of the breast early give very satisfactory results. True, not all lumps in the breast are malignant, but it is not safe to trust and hope till too late.

Finally, we may say that the object of this message has been to show, first, the rapid increase in frequency of cancer, especially in women, and, second, the imperative need for a more comprehensive conception of its earlier manifestations which are essential to an early diagnosis, which in turn is essential in the obtaining satisfactory results in operations for its extirpation. Shrinking from operations till too late are fatal. It is well understood that nothing but extirpation is warrantable, despite any claims to the contrary. Intelligent, courageous action on the part of women alone can reduce the mortality of cancer.

Desiring particularly to impress upon your mind the foregoing thoughts, we would, for emphasis, recapitulate what we have already said. Cancer of the uterus should be suspected at least whenever in a woman over 30 there is an increase in the quantity of the regular menstrual flow, or whenever there is the slightest bleeding outside or between the regular periods, or whenever there is an increase of a vaginal discharge without apparent reason. Especially are these manifestations significant when associated with a perceptible loss of flesh and a growing weakness, even though no pelvic lesions are discovered on examination. The appearance after the menopause of a bloody discharge, almost invariably means cancer. At least such a condition demands immediate investigation. We are often surprised by making a microscopical examination of tissue taken from a cervix to find evidences of beginning cancer, even though there may have been no symptoms present. Hence, it must be apparent that to make an early diagnosis people must be aroused.

It is claimed by excellent authority, in which we fully concur, that every woman who has had children and is over 30 years of age, who suffers from a chronic cervicitis with a leucorrhea, carries about in her what may be the beginning of cancer. It is obvious, then, that all chronic leucorrhea should be treated.

Once well begun, cancer spreads to adjacent tissues by continuity, slowly and by the lymphatic glands, both near and remote, making it impossible in advanced cases to remove all possibly infected glands.

Thus we see from every viewpoint that, if we are to check the ravages of this most terrible of scourges, we must awaken a keener appreciation of its early manifestations.

Furthermore, we find that it is not so much a fault of the profession as of the people that such cases are not earlier submitted for examination and cure. We would, therefore, present this as the most important message possible to the people.

After the above simple presentation of the subject to the people for their reflection, we may profitably, as a profession, go a little further, and I think I use the word little advisedly, as it, after all, is but little farther that we can go.

Referring to the excellent articles of Levin and Savidge, we find much of interest for our consideration. First. "Experimental research has proven that any normal cell, by the aid of an external irritant, can be transformed into a malignant cell, which, when introduced into one host, maintains its malignancy, while in another it loses it. The deduction is, therefore, that cancer is the result of the interaction between a certain external irritant and the constitutional reactivity of the individual. Unless, then, a uniform specific irritant causing cancer of a parasitic nature is found, and present observations do not indicate it, we have to search each individual case for a different irritant and also different constitutional conditions within the subject. As, for example, certain irritants produce cancer in a limited number, while others similarly exposed are immune."

The cervix uteri, as we have assumed, is the site of the majority of cancers, due, we have claimed, to the trauma sustained at childbirth. For this reason, repair is urged as a prophylactic measure.

"In cancer of the body of the uterus, such irritating cause does not obtain, yet cancer occurs in this region, though not as often." Cullen found out of 19 cases of cancer of the body, 10 had never had children. Theilhaber ascribes the cause of cancer of the cervix to lacerations and subsequent anemia of the scar. While cancer of the body of the uterus, occurring at a more advanced age, is due to the general anemia of the uterus, cancer has occurred where the lacerations had been properly repaired. Levin shows by statistics that but a small per cent of lacerated cervices eventuate in cancer, and concludes that normal physiological conditions of menstruation, child-bearing and menopause hardly give rise to cancer, nor is the trauma incident to parturition sufficient to account for it.

It may be interesting to know that the Imperial Cancer Research Fund in seven years has studied 200,000 mice and has 60 different species of cancer growing, as stated in Savidge's paper. Seven years ago no one conceived the idea that cancer could be kept growing four times the life of the animal.

Every case has its remedial struggle, as Hodenpyle's case, with which you are familiar, had in her serum power of remedial force to cure her own cancer. In her case, condition overcame the cancer. Usually, the cancer overcomes the condition.

The resume of Savidge and authors consulted is: "Mouse cancer is analogous with human cancer. Cancer is a disease of age. Cancer cannot be inoculated upon the aged. Condition is vital in cancer, extending to some, complete immunity; to others, a rare self-cure; but, to the majority of the afflicted,, rhythmic attempt at self-cure, with final defeat. It bears no relation to kind of diet; it is not hereditary; it is not contagious on infectious(?). Cancer must be grown, and its cell is differentiated according to the tissue from which it arises."

Savidge further observes: "It would seem, logically, that there must be a pre-cancer stage, a pre-existing condition, needing only the exciting cause to produce cancer, differing, it may be, in different individuals. He further takes hope for the future in the above statements—if cancer cannot be caught, cannot be inherited, but must be grown, and if its growth depends upon pre-cancer conditions plus a specific irritation, we have help for the community."

*Treatment.*—Prompt surgery may remove chronic irritations, ulcers, moles, benign tumors, etc., which Crile calls "potential cancers." Surgery must be early before cachexia shows. The unsuspecting 80,000, spoken of earlier in the paper, should know this.

Some of you doubtless received blanks from the Missouri State Medical Association, Collective Cancer Investigation, to be filled in, giving various data for the purpose of tabulated study and the observation of any particular feature that may appear often enough to attract attention. We should faithfully report all cases of cancer, thus aiding a widespread effort.

Notwithstanding Savidge's statement that cancer is not contagious or infectious, we find much which would seem to indicate the contrary. In the last *American Journal of Obstetrics*, etc., J. Garland Sherrill, of Louisville, Ky., cites cases having been reported that go to show quite conclusively that cancer is, or may be, contracted; as, for example, in one place in Germany it occurred in almost every house—in truth, a cancer lane. A man gave nutrient clysters daily for six months with cancer of the rectum; afterward took with cancer of the lower lip. It was removed, but returned in a year and a half. While he was sick his wife took down with cancer of the breast. No hereditary tendency in either case discoverable. A young woman whose mother died of an intestinal cancer, and an aunt on the mother's side, used a clyster tube which the mother had discarded. In six months, she developed intestinal cancer. Another case communicated by means of a pipe. Two similar cases, one cited by Dives and one in which a woman contracted cancer by washing clothes contaminated by her husband, who had cancer. Another case in which cancer of the penis developed from cancer of the uterus. Rare as this may seem, 36 such cases have been reported. Ebert collected 22 cases in which a direct transfer took place in the same individual, as from lip to lip, tongue to gums, or tongue to palate—cases of infection of physicians by inoculation. Budd has seen five die in cancer hospitals in ten years. Thus we are confronted by conflicting opinions all along the line, so that we can truly say, as yet, we know little of the nature, and less of the cause of carcinoma.—*Medical Herald.*

